# Cerebrospinal Fluid Concentrations of Neuronal Proteins Are Reduced in Primary Angiitis of the Central Nervous System

**DOI:** 10.3389/fneur.2018.00407

**Published:** 2018-06-05

**Authors:** Tillmann Ruland, Jolien Wolbert, Michael G. Gottschalk, Simone König, Andreas Schulte-Mecklenbeck, Jens Minnerup, Sven G. Meuth, Catharina C. Groß, Heinz Wiendl, Gerd Meyer zu Hörste

**Affiliations:** ^1^Department of Neurology, University of Münster, Münster, Germany; ^2^Department of Psychiatry and Psychotherapy, University of Münster, Münster, Germany; ^3^Department of Psychiatry and Psychotherapy, Medical Center - University of Freiburg, Faculty of Medicine, University of Freiburg, Freiburg, Germany; ^4^Core Unit Proteomics, Interdisciplinary Center for Clinical Research, University of Münster, Münster, Germany

**Keywords:** PACNS, CSF, mass spectrometry, flow cytometry, amyloid-beta A4 protein

## Abstract

Primary angiitis of the central nervous system (PACNS) is a rare autoimmune vasculitis limited to the CNS often causing substantial disability. Understanding of this disease is impaired by the lack of available biomaterial. Here, we collected cerebrospinal fluid (CSF) from patients with PACNS and matched controls and performed unbiased proteomics profiling using ion mobility mass spectrometry to identify novel disease mechanisms and candidate biomarkers. We identified 14 candidate proteins, including amyloid-beta A4 protein (APP), with reduced abundance in the CSF of PACNS patients and validated APP by Enzyme-linked Immunosorbent Assay (ELISA) in an extended cohort of patients with PACNS. Subsequent functional annotation surprisingly suggested neuronal pathology rather than immune activation in PACNS. Our study is the first to employ mass spectrometry to local immune reactions in PACNS and it identifies candidates such as APP with pathogenic relevance in PACNS to improve patient care in the future.

## Introduction

Primary angiitis of the central nervous system (PACNS) is a rare form of vasculitis with approximately 700 reported cases published in the literature worldwide (1). PACNS is generally considered to be an autoimmune disease of the vasculature limited to vessels of the central nervous system (CNS). The pathogenesis of PACNS is poorly understood and therapy design is often challenging. Management of PACNS would be facilitated by improved diagnostic or prognostic biomarkers and mechanistic understanding of the disease. Unbiased screening approaches as a strategic could serve as the conceptual basis for subsequent mechanistic studies.

The median age at onset of PACNS is around 50 years and men are more often affected then women. Clinical presentation displays a considerable heterogeneity. Initially, PACNS patients may present with unspecific symptoms such as headache and encephalopathy. Fever, seizures or focal symptoms are less common, but can also occur. Moreover, strokes, transient ischaemic attacks, and hemorrhages occur in 30–50% of patients (2). Very little is known about the pathomechanisms of PACNS. Due to its clinical heterogeneity, different molecular subtypes of PACNS have been proposed encompassing granulomatous angiitis of the CNS and amyloid-β-related cerebral angiitis (ABRA) (2).

Histopathological studies have shown three distinct morphological patterns: acute necrotizing, purely lymphocytic, and granulomatous vasculitis (3). Histologically acute neutrophilic inflammation is the main diagnostic feature in active necrotizing vasculitis. Lymphocytic vasculitis features infiltration of B cells and granulomatous vasculitis is characterized by perivascular infiltration of mononuclear and granulomatous as well as multinucleated giant cells. Recent studies have claimed PACNS to be a CD4^+^ T cell-mediated disease. In fact, the CSF of a patient with ABRA contained a substantial fraction of activated CD69^+^ CD4^+^ T cells, even remote from the primary site of inflammation. CD4^+^ T cells were present in close proximity to MHC class II–expressing epithelioid macrophages and multinucleated giant cells (4). Furthermore, CD4^+^ T cells in PACNS produce significantly more pro-inflammatory interleukin-17 compared to ischemic stroke controls (5). Moreover, in ABRA CD68^+^ macrophages extend to the parenchyma surrounding the vessel walls (6). Nevertheless, the cause of PACNS remains unknown.

Present diagnostic criteria of PACNS include (1) the presence of an acquired otherwise unexplained neurological or psychiatric deficit, (2) the presence of either classic angiographic or histopathological features of angiitis within the CNS, and (3) no evidence of systemic vasculitis or any disorder that could cause or mimic the angiographic or pathological features of the disease (7).

Conventional CSF analysis in PACNS usually features lymphocytic pleocytosis and elevated protein levels and can thus aid in the diagnosis of PACNS. Magnetic resonance (MR) imaging can reveal ischemic and hemorrhagic lesions in cortical, subcortical, and deep gray matter structures (8). CNS angiography shows alterations of blood vessel irregularities such as stenosis and dilatation. Indirect non-invasive imaging angiography such as computed tomography (CT)- or MR-angiography constitute alternatives to invasive CNS angiography. Recent advances in high resolution MR imaging of the vessel wall, such as black-blood contrast-enhanced *T*1 weighted images, may also be useful in the assessment of PACNS (9–12). Finally, brain biopsy provides further important histopathological evidence for PACNS. However, none of the diagnostic procedures provide an easily quantifiable laboratory surrogate marker.

In summary, due to the small number of cases and limited amount of available biomaterial (e.g., blood, CSF, biopsy specimen) it has been difficult to identify molecular mechanisms to better understand the immunological signatures of PACNS and to articulate hypotheses regarding pathomechanisms. High throughput and unbiased proteomics may provide new molecular insights in rare diseases (13–15). Mass-spectrometry-based techniques allow determining the relative abundance of a considerable fraction of the proteome without a pre-selection of target proteins. Recent studies in neuropsychiatric disorders and cancer serve as an example and identified potential diagnostic and prognostic markers in serum (16, 17).

Here, we analyzed CSF cells by flow cytometry and non-cellular CSF using ultrahigh-definition mass spectrometry followed by multivariate statistics in patients diagnosed with PACNS in comparison to a control group (18). Functional annotation network analysis was performed to identify relevant protein interactions and derive information of potentially affected biological pathways. Surprisingly, we find that APP, as validated by Enzyme-linked Immunosorbent Assay (ELISA), and other proteins of neuronal origin are less abundant in the CSF of PACNS patients suggesting a potential link between inflammation and neurodegeneration in this primary vasculitis as a conceptual basis for further mechanistic studies.

## Materials and methods

### Study participants and sample collection

**Figure 1:**
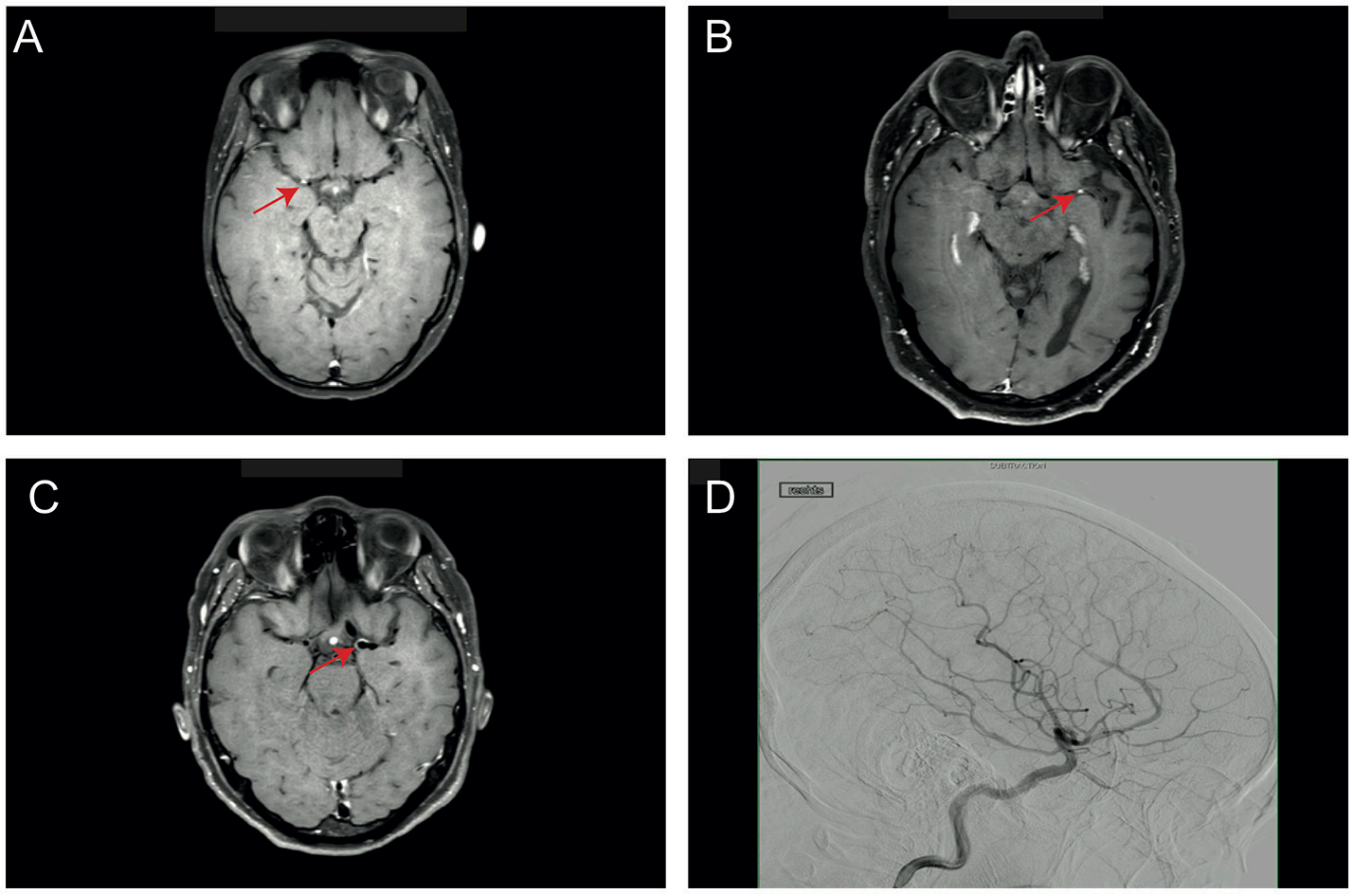
Representative imaging findings in PACNS patients. **(A–C)** Images of patients 1–3 with black-blood contrast-enhanced *T*1 weighted sequences, which show contrast enhancement in the vessel walls (red arrow). **(D)** Angiography image of PACNS patient 4 with longest duration of diseases showing pathognomonic blood vessel irregularities such as stenosis.

This study was approved by the ethics committee of the medical association Westphalia-Lippe, Germany (2016-053-f-S). Four patients diagnosed with PACNS were included. Diagnosis was based on clinical symptoms, MRI and angiography findings and after having ruled out systemic vasculitis. The imaging diagnosis of PACNS was based on digital subtraction angiography and MR imaging using black-blood contrast-enhanced *T*1 weighted sequences, which showed contrast enhancement in the vessel walls (see Figure 1). Four patients diagnosed with idiopathic intracranial hypertension (IIH) were chosen as matched controls (see Tables 1, 2 for details). Matching was performed for age, gender and BMI. Blood and CSF of all patients were routinely analyzed to rule out autoimmune, infectious, or malignant disease (see Table 2 for details).

**Table 1:** Patient demographic and clinical characteristics.

**Diagnosis**	**Age**	**BMI**	**Smoking**	**Comorbidities**	**Date of diagnosis**	**Date of sampling**	**Immunotherapy**	**PACNS specific changes in MRI + MRA**	**PACNS specific changes in angiography**	**CNS biopsy**
PACNS	20–25	19.4	No	None	May 16	May 16	Naive	Yes	Yes	No
PACNS	50–55	25.7	No	Diabetes mellitus type II, arterial hypertension	May 12	Nov 16	Yes^*^	Yes	Yes	Yes
PACNS	45–50	30.1	No	Arterial hypertension	Apr 16	Apr 16	Naive	Yes	Yes	No
PACNS	45–50	37.9	No	Diabetes mellitus type II, arterial hypertension	Sep 16	Mar 15	Naive	Yes	Yes	No
IIH	25–30	34.5	No	Arterial hypertension	Oct 15	Oct 15		No		
IIH	40–45	33.9	No	None	Nov 15	Jan 16		No		
IIH	40–45	35.5	No	None	May 15	May 15		No		
IIH	75–80	31.2	No	None	Nov 15	Dec 15		No		

*No significant difference for key clinical features such as age (p = 0.88, Mann-Whitney-U-Test), gender (p = 0.47, Fisher's exact test) or body mass index (p = 0.34, Mann-Whitney-U-Test) were found. PACNS specific changes in MRI/MRA and angiography included blood vessel irregularities such as stenosis and dilatation, infarction and black blood contrast enhanced T1 weighted images showing contrast enhancement in the vessel walls (Figure 1)*.

* *Immunotherapy with 16 cycles of cyclophosphamid (750 mg/m^2^ KOF) June 2012 to Feb 2013 and May 2015 to Nov 2016, methotrexat from Feb 2013 to Oct 2013. CNS, central nervous system; PACNS, primary angiitis of the CNS; BMI, body mass index; MRI, magnetic resonance imaging; MRA, magnetic resonance angiography*.

**Table 2:** CSF and blood tests for the PACNS and IHH groups.

**Diagnosis**	**Total protein (mg/l)**	**Total cell count # cells**	**Lympocytes # cells**	**Oligoclonal bands Typ**	**Viral infection**	**Hepatitis**	**Autoimmune disease**	**Treponema pallidum test**	**Borrelia test**
PACNS	177	3	3	1	Negative	Negative	Negative	Negative	Negative
PACNS	468	0	0	4	Negative	Negative	Negative	Negative	Negative
PACNS	794	2	2	1	Negative	Negative	Negative	Negative	Negative
PACNS	634	1	1	4	Not available	Negative	Negative	Not available	Not available
IIH	152	1	1	1					
IIH	215	0	0	1					
IIH	238	2	2	1					
IIH	594	0	0	1					

*Extended CSF and blood test to rule out infection or autoimmune disease was done for PACNS only. Oligoclonal bands Typ 1 are defined by no immunglobulin G bands in CSF or blood; Typ 4 immunglobulin G bands in CSF and blood point toward a deficiency in the blood-brain barrier. Tests for viral infection included CMV, EBV, HHV-6, HSV-1, HSV-2, and VZV PCR in CSF. Test for hepatitis included anti-HAV IgM., HBsAg.; anti-HBc and anti-HCV. Autoimmune disease tests included Rheumatoid factor, Waaler Rose Test, anti cyclic citrullinated peptide (CCP), antinuclear antibody (ANA), anti-dsDNA, antineutrophil cytoplasmic antibodies (ANCA). For Treponema pallidum detection CSF and blood were tested by the Treponema pallidum hemagglutination assay (TPHA) and a chemiluminescence assay (CLIA). For Borrelia burgdorferi detection CSF and blood were tested with an Enzyme-linked Immunosorbent Assay (ELISA)*.

The patients were followed-up in our department for at least 9 months after diagnosis was made. During this period consecutive MR imaging was performed and due to the diseases activity immunosuppressant therapy was started in all patients.

In addition, all CSF cell samples and matching blood samples were analyzed by flow cytometry using a predefined staining panel (see below) and the non-cellular CSF fraction was processed for consecutive proteome analysis. For this, CSF samples were centrifuged at 350 g for 10 min, the CSF supernatant was removed and stored at −80°C until further proteomics processing. Maximum storage time for any sample was 21 months with an average of 11.38 ± 5.50 months. For ELISA analysis an additional four patients diagnosed as described above as well as four controls with IIH were selected from our biobank (see Supplementary Table S4 for details).

### Flow cytometry

CSF and EDTA blood samples were obtained from all patients and processed as described previously (19). Briefly, cells from CSF or 100 μl EDTA blood were incubated in VersaLyse buffer (Beckman Coulter; Brea, CA) for 10 min and subsequently washed three times with PBS supplemented with 2% heat-inactivated FCS and 2 mM EDTA. Following incubation with fluorochrome-conjugated antibodies (CD14-FITC, CD138-PE, HLA-DR-ECD, CD3-PC5.5, CD56-PC7, CD4-APC, CD19-APCAlexafluor700, CD16-APCAlexafluor750, CD8-PacificBlue, and CD45-KromeOrange, all Beckman Coulter), cells were acquired on a Navios flow cytometer (Beckman Coulter). Analysis was conducted with Kaluza V1.2. Lymphocytes, monocytes, and granulocytes were selected based on forwards scatter channel, sideward scatter channel, CD14, and CD45 expression characteristics. Lymphocytes subsets were selected as CD3^+^CD4^+^ (T helper cells), CD3^+^CD8^+^ (cytotoxic T cells), CD3^+^HLA-DR^+^ (activated T cells), CD3^−^CD56^+^ (NK cells), CD3^−^CD19^+^ (B cells), CD3^−^CD19^+^CD138^+^ (plasma cells), whereas monocyte subsets were selected as CD14^+^CD16^−^ (classical monocytes), CD14^+^CD16^+^ (non-classical monocytes) cells.

### Liquid-chromatography ultra-high definition mass spectrometry (LC-UDMSe) analysis

For proteome analysis, CSF samples (1 ml each) were depleted using a *ProteoMiner Small Capacity Protein Enrichment Kit* from Bio-Rad (Hercules, CA) according to the instructions of the manufacturer. For the second depletion, the eluate of the first depletion was re-buffered using Zeba-Spin desalting columns 7 kD (ThermoFisher) and the ProteoMiner beads were washed using 20% ethanol. The second depletion then proceeded in the same way as the first.

On filter-digestion and liquid-chromatography mass spectrometry (LC-MS) was performed as described previously (18). Briefly, the protein solution was transferred to Nanosep Omega filter units (10 kDa MWCO, Pall). The sample on the filter was rinsed by centrifuging (14,000 × g, 4 °C, 15 min) with urea buffer (100 μl, 8 M urea, 100 mM Tris Base, pH 7.5). For reduction, the sample was vortexed with 100 ml urea buffer containing 50 mM DTT for 30 min followed by centrifuging and washing with urea buffer as above. Alkylation proceeded for 25 min in urea buffer containing 50 mM iodoacetamide (100 μl) on a vortexer in the dark followed by centrifuging and incubation with 50 mM DTT containing urea buffer (100 μl) for 15 min. Subsequently, the filter unit was rinsed with 100 μl urea buffer followed by five times washing with 100 μl 50 mM ammonium bicarbonate in 5% acetonitrile. For digestion, 40 μl trypsin solution (40 ng/μl in 5% acetonitrile) and 60 μl mass spectrometry-grade water were added. The filter unit was covered with parafilm and tryptic digestion proceeded at 37°C overnight. Peptides were collected by centrifuging three times with 100 μl 0.1% formic acid containing 5% acetonitrile; all solutions were pooled and dried. For analysis, peptides were re-dissolved in 100 μl of the same solution. High-definition MS was performed using Synapt G2 Si ion mobility mass spectrometer coupled to M-Class ultra-performance liquid chromatography (UPLC) (Waters Corp.) with a 90 min gradient (solvent system 100% water vs. 100% acetonitrile, both containing 0.1% formic acid; trap column V/M Symmetry C18 100 Å 5 μm, 180 μm × 20 mm; reversed phase column HSS T3 1.8 μm 75 μm × 200 mm;0.5 μl injection volume).

### Enzyme-linked immunosorbent assay (ELISA)

APP concentrations in the undepleted CSF samples were measured using a Human APP DuoSet ELISA kit from R&D Systems, Minneapolis USA, according to the manufacturer's instructions with a minimum detection limit of 0.625 ng/ml and using a protein standard curve for quantification (see Supplementary Table S5 for details).

### Statistical analysis of LC-UDMSe data

Data was pre-processed using Progenesis QI proteomics software (Nonlinear Dynamics, Version 2.0) using the UniProt human database (downloaded October 2015) generating normalized protein abundances for each sample.

Statistical analyses were additionally performed in R (http://www.R-project.org/) (20). The Reproducibility Optimized Test Statistic (ROTS) R package version 1.6.0 was used for statistical analysis. ROTS aims to rank genomic features of interest (such as genes, proteins and transcripts) in order of evidence for differential expression in two-group comparisons. It uses a data adaptive method, which can optimize its parameters based on intrinsic features of input data. False discovery rate (FDR) was set below a value of 0.05 (21, 22). To increase confidence only proteins with ≥1 unique peptide were considered for further analysis and were reported. A unique peptide is defined as a peptide, which based on its molecular properties, only exists in one protein and is therefore specific (23).

### Network analysis

A list of all proteins with fold changes found to be significantly different in the vasculitis vs. IIH comparison were uploaded to http://www.networkanalyst.ca/faces/home.xhtml. Using this online resource, a IMEx interactome based on the InnateDB platform was created (24). The network is generated by first mapping the significant proteins to the underlying protein-protein interaction (PPI) database. A search algorithm is then performed to identify first-order neighbors (proteins that directly interact with a given protein) for each of these mapped proteins. The resulting nodes and their interaction partners are returned to build a subnetwork. Subsequently, a minimum network, which consisted of proteins directly interacting with each other, was chosen to study key nodes of functional connectivity (25, 26).

### Statistical analysis of flow cytometry and ELISA data

After testing for homogeneity of variance and normal distribution flow cytometry data was analyzed using Mann-Whitney-U-Tests. The ELISA results were analyzed using a Student's *t*-test. Results were considered significant at a *p*-value threshold of < 0.05.

## Results

We retrospectively identified four patients with PACNS from our inpatient clinic (Table 1), who were diagnosed based on clinical symptoms, MRI and angiography findings and after having ruled out systemic vasculitis (Table 2). We used four patients with IIH as control patients. All CSF samples had been analyzed by standard techniques. CSF supernatants had been collected and stored from all patients in a standardized fashion (see methods).

We compared demographic and clinical variables between groups (Tables 1, 2) and found no significant difference for age (*p* = 0.88), gender (*p* = 0.47), and body mass index (*p* = 0.34). Only one patient had been previously treated with two immunosuppressants and diagnosis had been established 54 months before sampling (see Table 1). For the other patients sampling was done at the time of PACNS diagnosis. One patient displayed symptoms of PACNS *before* diagnosis was made and sampling was performed at the onset of symptoms but *before* the diagnosis was established subsequently (Table 1).

**Figure 2:**
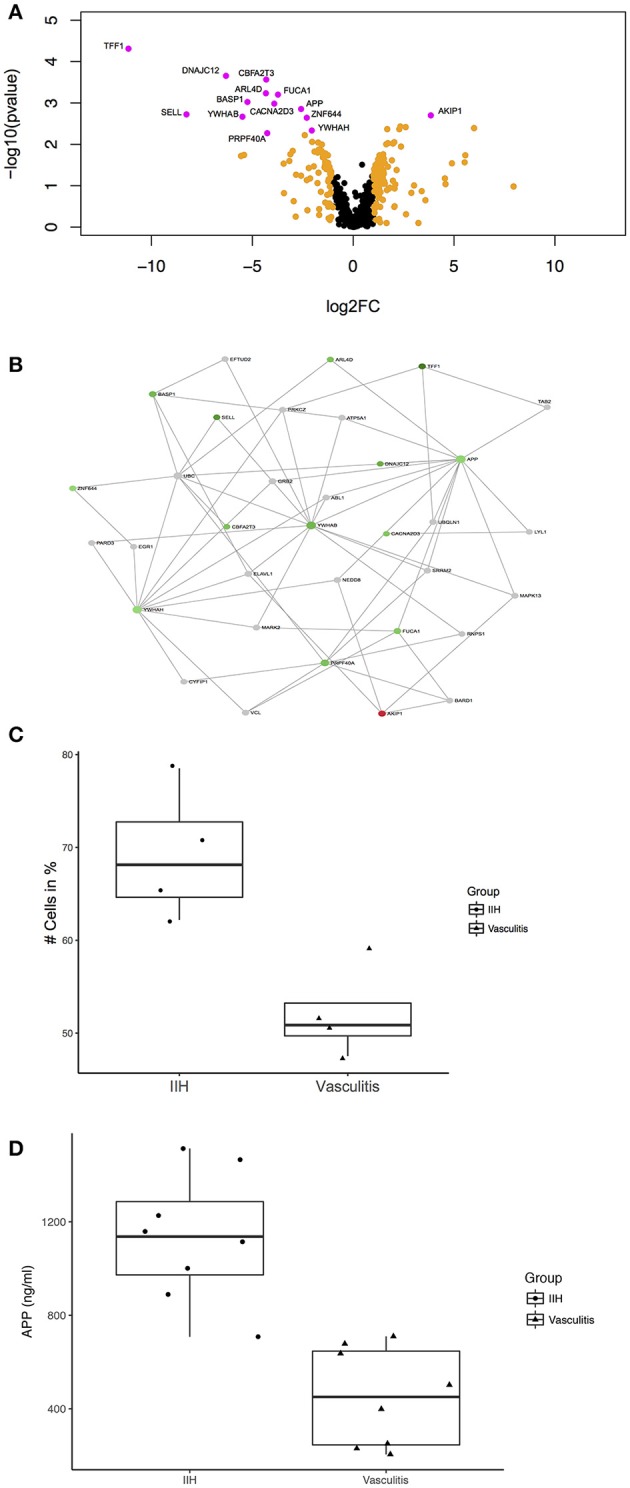
Unbiased proteomics identifies proteins with differential abundance in CSF in PACNS. **(A)** Volcano plot showing all identified proteins and highlighting proteins with differential abundance. Proteins with log2(fold change) >1 are highlighted in orange and proteins with an adjusted *p*-value ≤ 0.05 and a log2(fold change) >1 are highlighted in magenta comparing PACNS vs. IIH samples. Names of proteins with significantly different abundance are provided in the figure. **(B)** Network of proteins with an adjusted *p*-value ≤ 0.05 built with NetworkAnalyst. The network shows the proteins identified in our LC-UDMSe analysis as well as proteins that directly interact with a given protein to study key nodes of functional connectivity. Red color indicates upregulated, green color downregulated proteins based on fold change. **(C)** Boxplot of the percentage of CD3+ T cells in peripheral blood for PACNS vs. IIH samples (*p* = 0.029). **(D)** Boxplot of the concentrations of APP in CSF of PACNS vs. IHH samples (*p* = 6.41E-05).

We collected data of flow cytometry performed using cells from CSF and blood from all 8 patients in our study. Patients with PACNS exhibited a reduced percentage of CD3^+^ T cells in the peripheral blood compared to IIH patients (*p* = 0.029, Figure 2C) but did not show any other alterations in the analyzed populations in blood or CSF (Supplementary Table S2), which suggests that in our small sized cohort CSF and blood do not exhibit distinct alterations in cell composition in PACNS. We therefore speculated that non-cellular immune mechanisms could also be relevant in PACNS. To identify such potential non-cellular changes in the CSF, we performed unbiased, mass spectrometry of the non-cellular fraction of CSF from PACNS and IIH patients.

First we depleted CSF samples of most abundant proteins to improve the sensitivity for detecting low abundance proteins. Then we performed non-hypothesis driven label-free liquid-chromatography ultra-high definition mass spectrometry (LC-UDMSe) analysis comparing the CSF of four patients with PACNS to four controls with IIH. We detected 591 quantifiable proteins in all CSF samples (Supplementary Table S1). We then tested for differential abundance between IIH and PACNS CSF samples and identified 95 proteins with differential abundance in PACNS-derived CSF (*p* < 0.05). To reduce the false positive rate, we applied very stringent selection criteria. After FDR correction, 14 proteins remained significantly different in PACNS (adjusted *p* < 0.05) (Figure 2A). Notably, the vast majority of proteins was less abundant in PACNS samples. Except for CD62L/L-selectin (encoded by *SELL*) detected proteins were related to neuronal or nervous system function rather than immune system function illustrated by proteins such as brain abundant membrane attached signal protein 1 (gene *BASP1*), amyloid beta A4 protein (gene *APP*), and 14-3-3 proteins (genes *YWHAB* and *YWHAH*). This indicates that our screening approach preferentially detected abundance changes in proteins indicative of nervous system damage rather than reflecting immune cell activation or infiltration into the CSF compartment. Compared to candidates identified by mass spectrometry analysis of CSF in multiple sclerosis (MS) and Alzheimer's disease (AD) as well as human brain extracellular fluids in acute stroke overlap was modest. Only APP was also discovered to be changed in MS and AD (27–29).

Next, we aimed to validate the findings of our unbiased screen using an additional protein quantification method. We therefore quantified APP protein concentration by ELISA in CSF samples of an extended cohort of PACNS and control patients. We added four additional patients diagnosed with PACNS and four controls to the samples analyzed by LC-UDMSe (Supplementary Tables S3, S4 for patients details). We found a significant decrease of APP concentrations in CSF in PACNS patients (451.44 ± 196.21 ng/ml) vs. controls (1513.7 ± 255.55 ng/ml); *t*_(14)_ = 5.61, *p* = 0.0000641 (Figure 2D). This supports the adequacy of our screening approach.

Next, we sought to embed proteins with differential abundance into a PPI network to aid our understanding of our findings. This PPI network analysis is based on publicly available evidence regarding the relationship of proteins. Some of this evidence includes physical PPI but does not prove interaction of proteins in a specific cell type (e.g., immune cells). By depicting a PPI network we rather aimed to put the identified proteins into cellular and functional context to search for important nodes (hubs), which might be useful as biomarkers (30). We identified the downregulated amyloid beta A4 protein (gene APP) [adjusted *p*-value < 0.05, log2 fold change (FC) = −2.59] as an important hub having several core connections in the newly defined network (Figure 2B). The network consisted of identified proteins as well as essential not detected proteins that maintained the network connection. Other notable candidate connections included Trefoil factor 1 (gene *TFF1*) (adj.*p* < 0.05, log2FC = −11.14), CD62L/L-selectin (gene *SELL*) (adj.*p* < 0.05, log2FC = −8.26), brain acid soluble protein 1 (gene *BASP1*) (adj.*p* < 0.05, log2FC = −5.25), 14-3-3 protein beta/alpha (gene *YWHAB*) (adj.*p* < 0.05, log2FC = −5.49) and 14-3-3 protein eta (gene *YWHAH*) (adj.*p* < 0.05, log2FC = −2.04). Therefore, changes of the CSF proteome in PACNS might reflect secondary nervous system pathology rather than immune activation. Nevertheless, these changes differ distinctly from findings in neurodegenerative disease (28, 31).

## Discussion

The objective of this study was to generate novel hypotheses regarding the molecular mechanisms driving PACNS—a rare but particularly disabling neurological disorder. We describe the first CSF-focussed unbiased proteomics screening approach to study local immunity in the vicinity of the CNS. In fact, we identified a number of proteins with differential abundance in PACNS (Figure 2A, Supplementary Table S1). Interesting candidates were APP and TFF1, SELL, BASP1, YWHAB, and YWHAH. Furthermore, APP could be validated using an additional protein quantification method in an extended cohort of patients supporting our technical approach and the potential relevance of APP in the pathogenesis of PACNS. All these proteins and the subsequent network analysis suggested that CSF in PACNS may reflect nervous system pathology rather than immune activation as significantly changed protein abundances were linked to neuronal rather than immune cell function. Based on these findings, we speculate that CSF markers of neuronal injury could be a surprisingly promising surrogate marker of CNS injury in PACNS if confirmed in subsequent studies. This study thus achieved its primary goal of articulating novel hypotheses in PACNS.

We performed down-stream analysis building a PPI network based on published evidence. This emphasized APP as central interaction hub and therefore potential key player in PACNS pathology. APP is one of the central proteins involved in CNS pathology in Alzheimer's disease (AD) (32). APP accumulates in extracellular plaques in AD and is cleaved by different secretases, which is thought to cause neuronal damage by disturbing signaling and inducing synaptotoxicity (33).

Studies concerning APP levels in AD have been inconsistent (34). A role of APP and its cleavage product beta-amyloid in the pathogenesis of other types of vasculopathy of the CNS has been reported previously (35). This has led to the proposal of a new subtype of CNS vasculopathy that has been named ABRA. Histopathologically ABRA was characterized by the presence of severe leptomeningeal and parenchymal amyloid angiopathy (36). Compared to our cohort with a mean age of 43 years, patients with ABRA in the above-mentioned study were generally older with a mean age of 67.3 years.

Downregulation of TFF1, which has been proposed as an anti-inflammatory agent in the brain, and downregulation of soluble L-Selectin having anti-inflammatory properties may reflect altered immune response in PACNS (37, 38). BASP1 has amyloid like properties for example by eliciting ion channels into membranes and is able to form fibril-like aggregates (39, 40). 14-3-3 proteins, on the other hand, control different cellular processes e.g., apoptosis. Deletion of YWHAH leads to severe disturbance of neuronal migration (41). During inflammatory signaling, suppression of YWHAH controls the induction of Toll-like receptor driven early pro-inflammatory cytokine and Type I interferon production (42) Furthermore, in large vessel vasculitides antibodies to 14-3-3 proteins were found pointing toward a roll of this protein family in PACNS (43).

Previous studies had shown that CD4^+^ T cells in the CSF of a patient with ABRA were increased (4), but this aspect had not been explored in PACNS. In the present study, we found no apparent differences in CSF cell composition by flow cytometry, which could reflect different mechanisms in ABRA and PACNS or may be due to the limited sample size of our cohort. We only found the percentage of CD3^+^ T cells in the blood to be decreased in PACNS vs. IIH controls which could be due to immunosuppressive treatment. The local activation of immune cells previously observed in biopsy studies, may therefore be reflected in the CSF (3).

Our present approach has limitations, which should be accounted for when interpreting the results. According to Calabrese and Mallek CNS tissue biopsy specimen is recommended for definite diagnosis of PACNS (1, 7). However, CNS biopsy is not performed in all patients and focussing only on biopsy-confirmed cases would have considerably limited available patient numbers and could have biased our study. Our study included patients who had been diagnosed based on angiography and black-blood contrast-enhanced *T*1 MRI images to confirm vessel abnormalities indicative of vasculitis, as well as CSF analysis to rule out infectious disease (9–12). This limitation could potentially result in inadvertently including patients with non-PACNS-related vessel alterations. We do, however, consider this possibility unlikely because we thoroughly selected and screened patients for differential diagnoses and all patients were repeatedly seen in our department after sampling. This long-term follow-up most likely excluded non-PACNS cases in our cohort.

The non-hypothesis driven proteomics approach of our study comes with inherent weaknesses. Our study does not provide mechanistic explanations for the reduction of neuronal protein abundance in the CSF in PACNS. Instead it provides the basis for better understanding the disease in the future. The ability to articulate new hypotheses is the key strength of such biomarker discovery studies.

Our study implies that CSF represents a unique window to better understand local immune reactions and neurodegenerative mechanisms in PACNS. If our findings were verified in larger cohorts and by additional techniques, it would allow confirming our list of candidates as potential diagnostic biomarkers. Future studies could also be designed to test for the prognostic values of our candidates. In conclusion, our findings suggest that APP and its possible interaction partners might be interesting candidate biomarkers to further investigate in patients with PACNS and that CSF could further help to diagnose PACNS in the future.

## Ethics statement

This study was carried out in accordance with the recommendations of the ethics committee of the medical association Westphalia-Lippe, Germany with written informed consent from all subjects. All subjects gave written informed consent in accordance with the Declaration of Helsinki. The protocol was approved by the ethics committee of the medical association Westphalia-Lippe, Germany (2016-053-f-S).

## Author contributions

TR and GMzH designed the study, recruited patients, performed statistical analyses, and wrote the manuscript. JW performed the ELISA and data analysis. SK performed the LC-UDMSe work including bioinformatic data analysis using Progenesis QIP. AS-M and CG performed the FACS work. TR and MG did the network as well as additional statistical analyses of the proteomic data. GMzH, SM, and HW supervised the study and were involved in its design. All authors contributed to and have approved the final manuscript.

### Conflict of interest statement

The authors declare that the research was conducted in the absence of any commercial or financial relationships that could be construed as a potential conflict of interest.

## Supplementary material

Supplementary Table S1 List of all identified proteins using ROTS.

Supplementary Table S2 Results of the flow cytometry of CSF and blood expressed as percentage of cells.

Supplementary Tables S3, S4 Patient demographics and clinical characteristics of the additional four PACNS and four IIH patients. (for details see additional document).

Supplementary Table S5 Results of the APP ELISA. Samples were run in duplicate.
