# Supplementary material for: Cerebrospinal Fluid Concentrations of Neuronal Proteins Are Reduced in Primary Angiitis of the Central Nervous System
Source: Front Neurol. 2018 Jun 5;9:407. doi: 10.3389/fneur.2018.00407 (PMC5996103; doi:10.3389/fneur.2018.00407)
Supplement: Supplementary file 4 [file Table_4.DOCX]

**Supplementary Table 3**

Legend to supplementary table 3: Patient demographic and clinical characteristics. No significant difference for key clinical features such as age (p = 0.34, *Mann-Whitney-U-Test*), gender (p = 0.49, *Fisher’s exact test*) or body mass index (p = 0.47, *Mann-Whitney-U-Test*) were found. PACNS specific changes in MRI/MRA and angiography included blood vessel irregularities such as stenosis and dilatation, infarction and black blood contrast enhanced T1 weighted images showing contrast enhancement in the vessel walls (Figure 2)

*Immunotherapy with 4 cycles of cyclophosphamid (750 mg/m² KOF). Abbreviation: Central nervous system (**CNS**), primary angiitis of the CNS (**PACNS**), body mass index (**BMI**), magnetic resonance imaging (**MRI**), magnetic resonance angiography (**MRA**),

| **Diagnosis** | **Age** | **BMI** | **Smoking** | **Comorbidities** | **Date of Diagnosis** | **Date of sampling** | **Immunotherapy** | **PACNS specific   changes  in MRI + MRA** | **PACNS specific  changes  in Angiography** | **CNS** |
| --- | --- | --- | --- | --- | --- | --- | --- | --- | --- | --- |
|  |  |  |  |  |  |  |  |  |  | **Biopsy** |
| PACNS | 55-60 | 25.70 | yes | none | March 11 | Oct 11 | Cyclophosphamid | yes | yes | yes |
| PACNS | 40-45 | 33.70 | no | Diabetes mellitus type II,  arterial hypertension | Nov 17 | Nov 17 | no | yes | yes | no |
| PACNS | 45-50 | 22.30 | yes | Major Depression | May 16 | May 16 | no | yes | yes | no |
| PACNS | 35-40 | 27.10 | no | Arterial hypertension,  primary hyperaldosteronism | Nov 16 | Nov 16 | no | yes | not available | no |
|  |  |  |  |  |  |  |  |  |  |  |
| IIH | 50-55 | not available | no | none | Jan 17 | Jan 17 |  | no |  |  |
| IIH | 30-35 | 27.9 | yes | none | March 17 | March 17 |  | no |  |  |
| IIH | 40-45 | 23.1 | no | hypothyroidism | June 17 | June 17 |  | no |  |  |
| IIH | 20-25 | 23.51 | no | none | Jan 17 | Jan 17 |  | no |  |  |

**Supplementary Table 4**

Legend to supplementary table 4: CSF and blood tests for the PACNS and IHH groups. Extended CSF and blood test to rule out infection or autoimmune disease was done for PACNS only. Oligoclonal bands Typ 1 are defined by no immunglobulin G bands in CSF or blood; Typ 4 immunglobulin G bands in CSF and blood point towards a deficiency in the blood-brain barrier. Tests for viral infection included CMV, EBV, HHV-6, HSV-1, HSV-2 and VZV PCR in CSF. Test for hepatitis included anti-HAV IgM., HBsAg.; anti-HBc and anti-HCV. Autoimmune disease tests included Rheumatoid factor, Waaler Rose Test, anti cyclic citrullinated peptide (CCP), antinuclear antibody (ANA), anti-dsDNA, antineutrophil cytoplasmic antibodies (ANCA). For Treponema pallidum detection CSF and blood were tested by the Treponema pallidum hemagglutination assay (TPHA) and a chemiluminescence assay (CLIA). For Borrelia burgdorferi detection CSF and blood were tested with an Enzyme-linked Immunosorbent Assay (ELISA).

| **Diagnosis** | **Total protein (mg/l)** | **Total cell count** | **Lympocytes** | **Oligoclonal bands** | **Viral infection** | **Hepatitis** | **Autoimmune Disease** | **Treponema pallidum Test** | **Borrelia Test** |
| --- | --- | --- | --- | --- | --- | --- | --- | --- | --- |
|  |  | **# cells** | **# cells** | **Typ** |  |  |  |  |  |
| PACNS | 477 | 2 | 2 | 1 | negative | negative | negative | negative | negative |
| PACNS | 675 | 3 | 3 | 1 | negative | negative | negative | negative | negative |
| PACNS | 573 | 2 | 2 | 4 | negative | negative | negative | negative | negative |
| PACNS | 713 | 15 | 13 | 1 | negative | negative | negative | negative | negative |
|  |  |  |  |  |  |  |  |  |  |
| IIH | 561 | 2 | 2 | 1 |  |  |  |  |  |
| IIH | 455 | 1 | 1 | 1 |  |  |  |  |  |
| IIH | 353 | 3 | 3 | 1 |  |  |  |  |  |
| IIH | 485 | 4 | 4 | 2 |  |  |  |  |  |
